# *Histoplasma capsulatum*: More Widespread than Previously Thought

**DOI:** 10.4269/ajtmh.14-0175

**Published:** 2014-06-04

**Authors:** Spinello Antinori

**Affiliations:** Department of Clinical and Biomedical Sciences “Luigi Sacco,” III Division of Infectious Diseases, University of Milano, Milano, Italy

Histoplasmosis is an endemic mycosis caused by a dimorphic fungus with two distinct varieties pathogenic for humans: *Histoplasma capsulatum* var. *capsulatum* and *H. capsulatum* var. *duboisii*. The latter is known to be restricted to sub-Saharan Africa, whereas the former is distributed worldwide. However, general textbooks of medical mycology when considering the geographic distribution of *H. capsulatum* var. *capsulatum* either refer only to “eastern United States (Ohio, Mississippi, and St. Lawrence River valleys) and most of Latin America,”^1^ or indicate that “isolated cases have been reported also from Southeast Asia and Africa.”^2^ In this issue of the *Journal*, Wang and others^3^ describe an autochthonous case of disseminated progressive histoplasmosis (DPH) observed in a young human immunodeficiency virus (HIV)-negative Chinese man. This report adds to the growing evidence from epidemiological surveys using histoplasmin skin tests,^4^ case reports,^5^ and a recent review of the literature^6^ (mostly of works written in Chinese) indicating that some areas of China should be included among those with medium-high endemicity for *H. capsulatum*. However, because China is one of the largest countries in the world, epidemiological information for clinicians should take into account which specific areas are involved. In this regard the histoplasmin skin test survey conducted by Zhao and others,^4^ showed an overall reactivity of 9.0% among 735 healthy volunteers and patients with lung diseases, with the highest prevalence observed in Hunan (8.9%) and Jiangsu (15.1%) provinces. In good agreement, in a review of 300 cases of histoplasmosis diagnosed in China (75% considered autochthonous),^6^ the geographical distribution of patients was 27.7% from Yunnan, 9.3% from Jiangsu and Hunan, 8.7% from Hubei and 7.3% from Sichuan. Another histoplasmin skin test survey conducted on 271 healthy students from Sichuan province showed a prevalence ranging from 6% to 35%, with the highest recorded in the southern part of the province.^7^

Overall, nearly 82% of all reviewed cases of histoplasmosis from China were reported from nine provinces through which the Yangtze River flows and where climate conditions are probably favorable for *H. capsulatum* growth^6^; it is worth noting that 86% of these cases were classified as disseminated, and in 52% of affected patients no underlying diseases were disclosed.

India is another Asian country where *H. capsulatum* is known to be endemic, although the true prevalence of this mycosis is still underappreciated. The first case was reported as early as 1954, and since then several cases (mostly DPH, even in the absence of underlying immunosuppression) have been published.^8^–^10^

In India the majority of histoplasmosis cases were reported from the eastern and north-eastern part of the country, especially from Calcutta (West Bengal) and Assam. Interestingly, as observed for the highly endemic areas in North America, both states are crossed by long rivers: the Ganges and the Brahamaputra, respectively.^8^–^10^ Moreover, the fungus was isolated from the soil of the Gangetic plains.^11^ A histoplasmin skin-test positivity rate of 12.3% was reported in northern India between the 1950s and 1970s.^12^

It has been hypothesized that in India a large number of cases might be unrecognized for a long period caused by low awareness of the disease and misdiagnose as tuberculosis or leishmaniasis.^8^,^10^ Oral cavity ulcers and bilateral adrenal enlargement seem to be particularly frequent among Indian patients, whereas skin lesions were observed in only 8% of cases.^8^,^10^ Also in the review of Chinese patients skin involvement was reported in a small fraction of cases (6.6%) and the authors speculated about the low number of HIV-positive patients in their file records.^6^ High rates of skin involvement have been observed among HIV-positive patients from South America and Africa.^13^

Thailand is another country of South-east Asia where localized foci of *H. capsulatum* exist, and the fungus has been shown by using nested polymerase chain reaction of soil contamined with bat guano and chicken droppings from Chiang Mai, a geographic area where *Penicillium marneffei* is also endemic.^14^ In contrast to the Chinese and Indian experiences, DPH in Thailand has been observed almost exclusively among HIV-infected patients, with 1,253 cases reported from 1984 to 2010 to the Ministry of Public Health.^14^

Based on sporadic case reports of autochthonous histoplasmosis, isolation of the fungus from soil in bat-infested caves and histoplasmin skin test surveys, Malaysia, Indonesia, Myanmar, and the Philippines should also be considered areas with pockets of endemicity for histoplasmosis.^15^,^16^ Interestingly, in a recent survey conducted in Europe, cases of disseminated histoplasmosis were diagnosed among elderly United Kingdom residents who had served in World War II in India and Myanmar and who did not leave their country of origin for more than 50 years after returning home.^17^

In Australia, cases of indigenous histoplasmosis were reported as early as 1948, and the organism has been cultured from fowl yards and caves, although the exact ecology is poorly understood^18^; the majority of cases were reported from the Queensland and northern New South Wales regions, which are characterized by tropical and subtropical climate. These regions are also crossed by long rivers (the Dumaresq and Macintyre rivers).

Outside Asia autochthonous cases of histoplasmosis have been reported sporadically from Italy,^13^,^17^ where studies conducted in the 1960s confirmed the presence of *H. capsulatum* in soil^19^ and animals,^20^ with the existence (confirmed with histoplasmin skin tests) of a pocket of endemicity along the Po River valley.^21^

*Histoplasma capsulatum* var. *duboisii*, characterized by a larger size (8–15 μm) and thicker walls than *H. capsulatum* var. *capsulatum*, is found in Madagascar and in central and western regions of sub-Saharan Africa (Malì, Chad, Niger, Nigeria, Democratic Republic of Congo, and Ghana). It is classically associated with skin, subcutaneous, and bone lesions, but disseminated disease has been described among HIV-positive patients^22^; because both pathogens coexist in Africa,^13^,^17^ it has been suggested that until the correct variety of the fungus has been identified African patients should not be described as affected by “African histoplasmosis.”^17^

In conclusion, our knowledge of the true worldwide distribution of *H. capsulatum* is still incomplete, and works such as that of Wang and coworkers are worthwhile. Improved access to diagnostic tests and increased awareness of the disease outside the well-known endemic areas will be helpful in redrawing the map of the geographic extent of this infection. Moreover, in an era of increasingly mobile people, physicians need to consider histoplasmosis in travelers and immigrants from the Indian subcontinent and South-east Asia in addition to regions traditionally considered endemic.
